# Preparation and Sustained-Release Performance of PLGA Microcapsule Carrier System

**DOI:** 10.3390/nano11071758

**Published:** 2021-07-06

**Authors:** Shuaikai Ren, Chunxin Wang, Liang Guo, Congcong Xu, Yan Wang, Changjiao Sun, Haixin Cui, Xiang Zhao

**Affiliations:** Institute of Environment and Sustainable Development in Agriculture, Chinese Academy of Agricultural Sciences, Beijing 100081, China; ren19801266562@163.com (S.R.); wangchunxin0213@163.com (C.W.); guoliang01@caas.cn (L.G.); boxiaoer@126.com (C.X.); wangyan03@caas.cn (Y.W.); sunchangjiao@caas.cn (C.S.); cuihaixin@caas.cn (H.C.)

**Keywords:** PLGA, microcapsule, encapsulation rate, sustained release

## Abstract

Microcapsules have been widely studied owing to their biocompatibility and potential for application in various areas, particularly drug delivery. However, the size of microcapsules is difficult to control, and the size distribution is very broad via various encapsulation techniques. Therefore, it is necessary to obtain microcapsules with uniform and tailored size for the construction of controlled-release drug carriers. In this study, emulsification and solvent evaporation methods were used to prepare a variety of ovalbumin-loaded poly (lactic-co-glycolic acid) (PLGA) microcapsules to determine the optimal preparation conditions. The particle size of the PLGA microcapsules prepared using the optimum conditions was approximately 200 nm, which showed good dispersibility with an ovalbumin encapsulation rate of more than 60%. In addition, porous microcapsules with different pore sizes were prepared by adding a varying amount of porogen bovine serum albumin (BSA) to the internal water phase. The release curve showed that the rate of protein release from the microcapsules could be controlled by adjusting the pore size. These findings demonstrated that we could tailor the morphology and structure of microcapsules by regulating the preparation conditions, thus controlling the encapsulation efficiency and the release performance of the microcapsule carrier system. We envision that this controlled-release novel microcapsule carrier system shows great potential for biomedical applications.

## 1. Introduction

Encapsulation technology refers to the process of encapsulating a substance in a suitable capsule material to form nano-, micro-, or millimeter-sized particles [1]. Numerous materials can be used for encapsulation and the choice of capsule material determines the physical and chemical properties of the capsule. Encapsulating an active material has the following advantages: The capsule material (1) keeps the active material from contacting the external environment and prevents its degradation and the loss of its activity [2]; (2) extends the half-life of the active material [3]; (3) allows sustained release [4]; and (4) reduces the evaporation and degradation of volatile substances [5]. Owing to these desirable properties, microcapsules have been widely used in pollutant adsorption, food quality preservation, stem cell culture and biomedicine [6,7,8,9]. It is important to prepare microcapsules with stable encapsulation and release efficiency using suitable methods [10]. Commonly used microcapsule preparation methods include the complex coacervation method, the ionic gel method, the microemulsion polymerization method, the layer-by-layer assembly method, and the air-suspension method [11,12,13,14,15]. Although many preparation technologies are suitable for microcapsules, no encapsulation method is suitable for all situations. The physical and chemical properties of the core and capsule materials, as well as the expected application of the product, dominate the choice of microcapsule technology [14].

Vectors commonly used in clinical practice are currently virus derived vectors. However, lentiviral and retroviral vectors, which are designed for insertion into the genome, introduce a high risk of gene disruption [16]. Therefore, non-viral vectors are considered to be promising alternatives. Microcapsule carriers can enhance the absorption of drugs, prolong the complex circulation time, provide sustained drug release, and improve therapeutic effects and safety [17]. In addition, the surface of carriers can be functionalized with antibodies, peptides or other biological molecules so that they are specifically recognized and bind to receptors expressed on the cell surface to achieve targeted drug delivery [18]. Numerous materials can be used for encapsulation, and the choice of capsule material determines the physical and chemical properties of the capsule. Important factors, such as the product requirements, environmental conditions, release characteristics and compatibility, must therefore be considered when choosing the encapsulation material. Polylactic acid (PLA) and poly(lactic-co-glycolic acid) (PLGA) are biocompatible, biodegradable, functional polymers with good encapsulation properties that can be metabolized in the body. Moreover, their strong plasticity, low price and versatility have enabled the development of various systems comprising PLA, PLGA, and their mixtures for biomedical applications, for example, as bio-carriers to control drug release in precision therapy and as scaffold materials for regenerative medicine [19,20,21,22]. Ren et al. loaded IL-1ra into PLGA microspheres, which significantly prolonged its half-life and produced a beneficial anti-inflammatory effect on macrophages. The carrier system was found to be effective against periodontitis [23]. Herrera et al. used the emulsification–solvent evaporation method to prepare ofloxacin and vancomycin-loaded PLA nanoparticles that resulted in strong antibacterial activity against Escherichia coli and methicillin-resistant Staphylococcus aureus, indicating their potential use as antibacterial agents [24]. Ghasemi synthesized three types of PLA nanoparticles by controlling the ratio of PLA and polyethyleneimine glycol. These nanoparticles exhibited good encapsulation efficiency and sustained-release of human growth hormone [25].

The particle sizes, structure and homogeneity of microcapsules affect their encapsulation rate, delivery behavior, absorption efficiency and release kinetics. A profound understanding of how these physicochemical properties regulate the fates and functions of microcapsules has become an important requirement. The optimization of preparation conditions for homogeneous microcapsules laid a foundation for their clinical applications. In this study, we developed a novel, biodegradable and controlled-release PLGA microcapsule carrier system via an emulsification and solvent evaporation method. The optimal conditions for the preparation of PLGA microcapsules and porous PLGA microcapsules were determined. The influence of factors, such as the volume ratio of the inner water phase to the oil phase, the volume ratio of the oil phase to the external water phase, the oil phase concentration, the external water phase concentration, and the surfactant concentration, on the morphology, particle size and dispersibility of the microcapsules were investigated. The morphologies of the microcapsules were observed with scanning electron microscopy, the particle size and dispersibility index of the microcapsules were measured with a nano laser particle sizer, and the drug loading and encapsulation efficiency of the microcapsules were determined with a BCA kit and microplate reader. The sustained release properties of PLGA microcapsules and porous PLGA microcapsules loaded with ovalbumin were further investigated. The release behaviors of ovalbumin could be precisely controlled by regulating the size and surface morphology of PLGA microcapsules. The prepared microcapsules that exhibited suitable particle size, size uniformity, good dispersibility, high ovalbumin encapsulation rate and sustained-release, are therefore expected to be appropriate carriers for protein drugs or vaccines in a wide range of applications. These results founded a theoretical basis for the future design of polymeric particle-based carrier systems.

## 2. Materials and Methods

### 2.1. Materials

Polyvinyl alcohol (PVA) was purchased from Johnson and Johnson Technology Co., Ltd. (Beijing, China), dichloromethane (DCM) was purchased from China Pharmaceutical Co., Ltd. (Shanghai, China), fetal bovine serum and ovalbumin were purchased from Gibco (Darmstadt, Germany). The unmodified mica flakes were purchased from Electron Microscopy China Co., Ltd. (Beijing, China). PLGA was purchased from Jinyue Biotechnology Company (Beijing, China). The BCA protein detection kit was purchased from Jiancheng Bio-Technology Co., Ltd. (Nanjing, China).

### 2.2. Methods

#### 2.2.1. Preparation of PLGA Microcapsules

We used the emulsification and solvent evaporation method to prepare PLGA microcapsules and explored the effects of varying the preparation conditions—including the PLGA concentration, phase ratios, and shear rate—on the microcapsule properties. Ovalbumin was dissolved in distilled water to obtain the internal water phase (20 mg/mL), PLGA was completely dissolved in DCM to obtain the oil phase (20 mg/mL, 40 mg/mL or 60 mg/mL), and PVA was dissolved in distilled water (stirring at 500 rpm and 80 °C) to obtain the external water phase (1 mg/mL, 3 mg/mL or 5 mg/mL). At a given shear rate (8000 rpm, 10,000 rpm or 12,000 rpm), the internal water phase was slowly added to the oil phase in given proportions to obtain a water-in-oil emulsion (internal water phase/oil phase = 1:3, 1:5 or 1:7). Then, the emulsion was poured into the external water phase with shearing. Shearing was continued for 5 min and then the sample was subjected to ultrasound in an ice bath for 3 min. The double emulsion was then placed in a fume hood and stirred overnight to allow the solvents to evaporate. After the DCM was completely evaporated, the obtained microcapsules were centrifuged at 10,000 rpm and the bottom layer was collected and dissolved in water. The centrifugation step was repeated three times, discarding the supernatant. The remaining sample was freeze-dried to obtain the ovalbumin-loaded microcapsules.

#### 2.2.2. Preparation of Porous PLGA Microcapsules

During the preparation of PLGA microcapsules, the addition of porogen BSA in the inner water phase was favorable for forming porous microcapsules. The ovalbumin (20 mg/mL) and an appropriate amount of BSA solution (10%, 15%, 20%) were added to distilled water to give the internal water phase. The PLGA was completely dissolved in methylene chloride to obtain the oil phase. PVA was dissolved in distilled water (stirring at 500 rpm and 80 °C) to obtain the external water phase (5 mg/mL). At the shear rate of 10,000 rpm, the internal water phase was slowly added to the oil phase to obtain a water-in-oil emulsion. The emulsion was then poured into the external water phase with shearing. Shearing was continued for 5 min and then the sample was subjected to ultrasound in an ice bath for 3 min. The double emulsion was then placed in a fume hood overnight with stirring to allow the solvents to evaporate. After the DCM had completely evaporated, the obtained microcapsules were centrifuged at 10,000 rpm, then the bottom layer was collected and dissolved in water. The centrifugation step was repeated three times, discarding the supernatant. The remaining sample was freeze-dried to obtain the ovalbumin-loaded microcapsules.

#### 2.2.3. Characterization of PLGA Microcapsules

The morphology of the PLGA microcapsules was observed using scanning electron microscopy. The freeze-dried microcapsules were suspended in water and dropped onto a silicon wafer and dried overnight. After coating with a thin layer of platinum using a sputtercoater (EM ACE600, Leica, Vienna, Austria), the samples were characterized by scanning electron microscope (SEM, HITACHI, Su-8010, Tokyo, Japan). The accelerating voltage used was 10.0 kV, and the current was 5.0 µA. A laser particle size analyzer (DLS Zetasizer Nano ZS90, Malvern Instruments, Worcestershire, UK) was used to measure the particle size and polydispersity index of the microcapsules at room temperature. PLGA microcapsules were diluted to a concentration of 1 µg/mL using distilled water. The final values were the average of three measurements.

#### 2.2.4. Determination of Encapsulation Efficiency

The encapsulation efficiency of the prepared microcapsules was determined using a BCA trace protein detection kit (Jiancheng Bio-Technology Co., Ltd., Nanjing, China). The sample solution was prepared as follows: A given quantity of freeze-dried microsphere powder was added to a distillation flask, DCM was added, and then the solution was evaporated to dryness at 40 °C. Distilled water was added to suspend the microcapsules and then the sample was filtered with a water membrane to remove impurities and was diluted to a volume of 25 mL with distilled water for later use. There were 3 blank wells (10 µL distilled water + 250 µL working solution) and 3 standard wells (10 µL standard solution + 250 µL working solution) in the 96-well plate, and the remaining wells were sample wells (10 µL sample solution + 250 µL working solution). The solution in each well was mixed and the absorbance at 562 nm was measured with a microplate reader (Thermo Fisher Scientifc, Inc., Waltham, MA, USA). Three replicates of each sample were measured. The formula for calculating the protein concentration in the sample was as follows:

(1) $$protein\;concentration=\frac{{\mathrm{OD}}_{m}-{\mathrm{OD}}_{b}}{{\mathrm{OD}}_{s}-{\mathrm{OD}}_{b}}\times standard\;product\;concentration\;\left(563\;µg/\mathrm{mL}\right).$$

(${\mathrm{OD}}_{m}$: measured OD value, ${\mathrm{OD}}_{b}$: blank OD value, ${\mathrm{OD}}_{s}$: standard OD value). Based on the measured protein concentration, the encapsulation efficiency of the microcapsules was obtained by conversion.

#### 2.2.5. Release Properties of the PLGA Microcapsules

The standard protein was dissolved in physiological saline to give solutions with concentrations of 0, 50, 100, 250, and 500 µg/mL. After shaking, the absorbance at 562 nm was measured to establish a standard curve.

A given quantity of microcapsules was placed into a dialysis bag with a molecular weight cut-off of 40,000–50,000 Da. After sealing, the bag was placed in a glass bottle filled with physiological saline and shaken at 37 °C and 100 rpm. At specific time points, 1 mL of the solution was removed for measurement, and 1 mL of physiological saline was added to maintain the volume. The release was monitored over 28 days. The concentration of ovalbumin in the sample at each time point was determined based on the standard curve, and the cumulative release curves of the microcapsules in vitro over time were established.

## 3. Results

### 3.1. Effect of PLGA Concentration on Microcapsule Morphology

**Figure 1:**
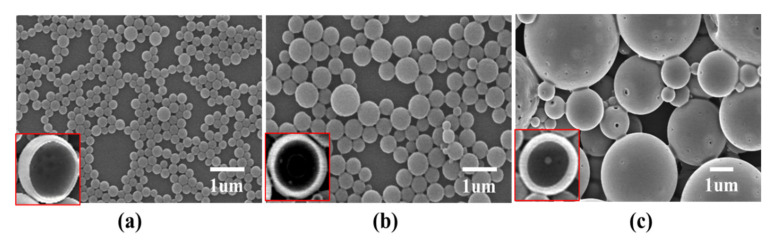
Effect of PLGA concentration on microcapsule morphology. PLGA concentrations of; (**a**) 20 mg/mL; (**b**) 40 mg/mL; and (**c**) 60 mg/mL.

To study the effect of the PLGA concentration on the morphology of microcapsules, the following PLGA microcapsule preparation conditions were investigated: concentration of ovalbumin in the internal aqueous phase, 20 mg/mL (the same concentration was used for the following experiment except where specifically mentioned); concentration of PLGA in the oil phase, 20, 40, or 60 mg/mL; volume ratio of the inner water phase to the oil phase, 1:5; concentration of PVA in the external water phase, 5 mg/mL; volume ratio of the oil phase to the external water phase, 1:5; and shear rate, 10,000 rpm. The results showed that the PLGA concentration influenced the prepared microcapsules’ particle size and uniformity (Figure 1); 20 mg/mL PLGA produced prepared PLGA microcapsules with a diameter of 245 nm and a thickness of the microcapsule wall of 25 nm, and all showed good uniformity. When the concentration was increased to 40 mg/mL, the particle size increased to 631 nm, the thickness of the microcapsule wall increased to 60 nm, and the uniformity was slightly reduced. When the concentration was increased to 60 mg/mL, the particle size and the thickness of the microcapsule wall were further increased and the uniformity decreased markedly (Table 1). Therefore, 20 mg/mL PLGA was chosen for the follow-up experiments. The results indicated that the higher concentrations of PLGA resulted in a higher viscosity of the oil phase and larger oil droplets dispersed in the water, thus causing the particle size and the thickness of the microsphere wall to increase.

### 3.2. Effect of Polyvinyl Alcohol Concentration on Microcapsule Morphology

**Figure 2:**
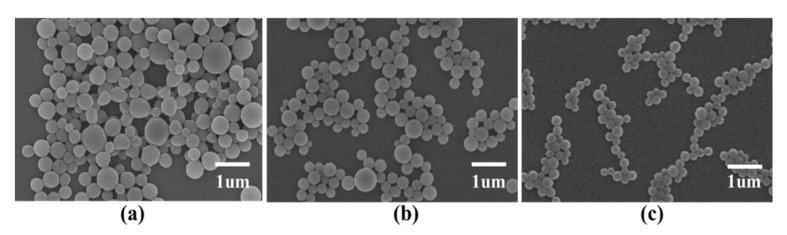
Effect of PVA concentration on microcapsule morphology. PVA concentrations of; (**a**) 1 mg/mL; (**b**) 3 mg/mL; and (**c**) 5 mg/mL.

To investigate the effect of PVA concentration on microcapsule morphology, the following PLGA microcapsule preparation conditions were used: volume ratio of the oil phase to the external water phase, 1:5; concentration of PVA in the external water phase, 1, 3, or 5 mg/mL; volume ratio of the oil phase to the external water phase, 1:5; and shear rate, 10,000 rpm. Results suggested that a higher PVA concentration led to a smoother surface and a smaller particle size of the PLGA microcapsules (Figure 2). When 1 mg/mL PVA was employed, the surfaces of the prepared PLGA microcapsules were rough and the particle size was 648 nm. When the concentration was increased to 3 mg/mL, there were fewer cracks on the surfaces, and the particle size decreased to 478 nm. When the concentration was increased to 5 mg/mL, the particle size of the PLGA microcapsules further decreased to 264 nm and the surface was smoother (Table 2). Thus, 5 mg/mL PVA was determined. PVA molecules can quickly adsorb on the oil–water interface, which effectively prevents emulsion drops’ aggregation. If the concentration of PVA is low, it is difficult to keep the stability of the emulsion droplet, resulting in a rough surface of the microcapsules.

### 3.3. Effect of the Volume Ratio of the Internal Water Phase to the Oil Phase on the Microcapsule Morphology

**Figure 3:**
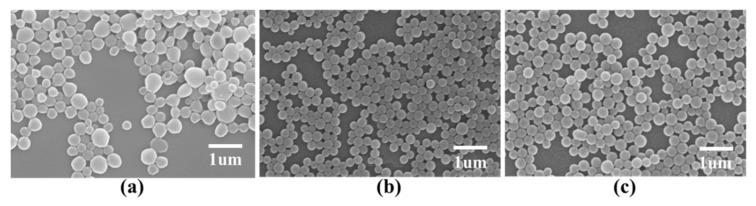
Effect of the volume ratio of the internal water phase to the oil phase on microcapsule morphology. Internal water phase/oil phase ratios of: (**a**) 1:3; (**b**) 1:5; and (**c**) 1:7.

The volume ratio of the internal water phase to the oil phase will affect the structure and encapsulation rate of the microcapsules and microspheres [26]. PLGA microcapsules were prepared under following conditions to explore the effect of the volume ratio of the internal water phase to the oil phase on the microcapsules’ morphology: the volume ratio of the inner water phase to the oil phase, 1:3, 1:5, or 1:7; PVA concentration of the outer water phase, 5 mg/mL; volume ratio of the oil phase to the outer water phase, 1:5; and shear rate, 10,000 rpm. The results indicated that both 1:5 and 1:7 volume ratios of the internal water phase to oil phase produced smaller and more uniform PLGA microcapsules (Figure 3). The 1:3 volume ratio resulted in a 458 nm average particle size of the prepared PLGA microcapsules. When the volume ratio of the oil phase was 1:5 or 1:7, the particle size was approximately 270 nm (Table 3), the surfaces were smooth, and the size uniformity was good.

### 3.4. Effect of the Volume Ratio of the Oil Phase to the External Water Phase on Microcapsule Morphology

**Figure 4:**
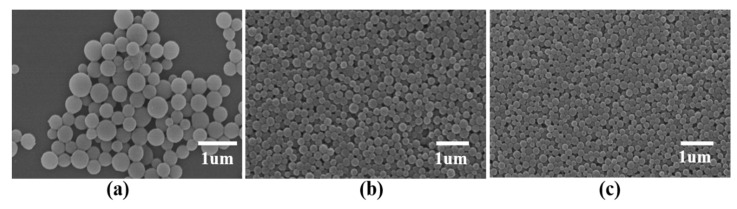
Effect of the volume ratio of the oil phase to the external water phase on the microcapsule morphology. Oil phase/external water phase of; (**a**) = 1:3; (**b**) 1:5; and (**c**) 1:7.

According to the result in 3.3, the 1:5 volume ratio of the inner water phase to the oil phase was chosen for studying the effect of the volume ratio of the oil phase to the external water phase on the microcapsules’ morphology. The 1:3, 1:5 or 1:7 volume ratios of the oil phase to the external water phase were tested, respectively, and the shear rate was 10,000 rpm. The results are shown in Figure 4 and Table 4. When the volume ratio of the oil phase to the external water phase was 1:3, the prepared microcapsules were polydispersed particles and showed agglomeration. When the volume ratio of the oil phase to the external water phase was 1:5 or 1:7, the particle size of the microcapsules decreased significantly, the dispersion was good, and the microcapsules were relatively uniform in size. This finding clearly indicates that we could tailor the morphology of microcapsules by regulating the volume ratio of the internal water phase to the oil phase and the volume ratio of the oil phase to the external water phase, thus controlling the encapsulation efficiency and the release performance of the microcapsule carrier system.

### 3.5. Effect of Shear Rate on Microcapsule Morphology

**Figure 5:**
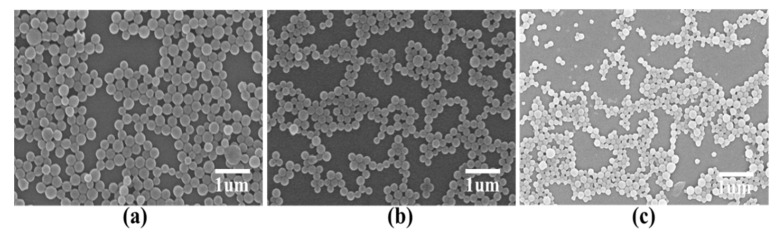
Effect of shear rate on the microcapsule morphology. Shear rates of: (**a**) 8000 rpm; (**b**) 10,000 rpm; and (**c**) 12,000 rpm.

To investigate the effect of shear rate on the microcapsule morphology, we used the following PLGA microcapsule preparation conditions: 8000, 10,000, or 12,000 rpm shear rate; volume ratio of the oil phase to the external water phase, 1:5. As the shear rate increased, the particle size of the prepared microcapsules decreased; however, when the rotation speed was increased to 12,000 rpm, pores appeared on the surfaces of the microcapsules and their shapes were deformed (Figure 5). A high shear rate makes it easier to obtain microspheres with a small particle size, but the microspheres are easily deformed (Table 5). Consequently, a 10,000 rpm shear rate was chosen for the following experiments.

### 3.6. Effect of the Volume Ratio of Each Phase on the Encapsulation Efficiency of PLGA Microcapsules

The particle size and uniformity of the ovalbumin-loaded PLGA microcapsules are important properties that affect their distribution in the body. Therefore, by investigating different preparation conditions, we determined that the optimum conditions for preparing PLGA microcapsules with a small particle size, good dispersion, and size uniformity were as follows: concentration of ovalbumin in the internal water phase, 20 mg/mL; concentration of PLGA in the oil phase, 20 mg/mL; volume ratio of the internal water phase to the oil phase, 1:5 or 1:7; volume ratio of the oil phase to the external water phase, 1:5 or 1:7; PVA concentration of the external water phase, 5 mg/mL; shear rate, 10,000 rpm. Of these conditions, the volume ratios of the inner water phase, oil phase, and external water phase were particularly important as they determined the encapsulation efficiency of the PLGA microcapsules. Therefore, we further investigated microcapsules with a volume ratio of the inner water phase to the oil phase of 1:5 and 1:7; and a volume ratio of the oil phase to the external water phase of 1:5 and 1:7. PLGA microcapsules were prepared using four different sets of conditions and the encapsulation efficiency of the four microcapsule varieties were measured using a BCA kit. The results are shown in Table 6. The findings showed that the encapsulation efficiency of the microcapsules was most greatly affected by the ratio of the internal water phase to the oil phase and, within a certain range, the higher the ratio of the oil phase, the greater the encapsulation efficiency of the microcapsules. The ratio of the oil phase to external water had a relatively small effect on the encapsulation efficiency of the microcapsules. This may be because when the amount of the oil phase was sufficient, the viscosity of the emulsion was high and the resistance of the drug to the external conditions was high, so the amount of migration was reduced, which would be conducive to encapsulation [27].

### 3.7. Effect of Different Phase Volume Ratios on the Sustained Release Performance of PLGA Microcapsules

**Figure 6:**
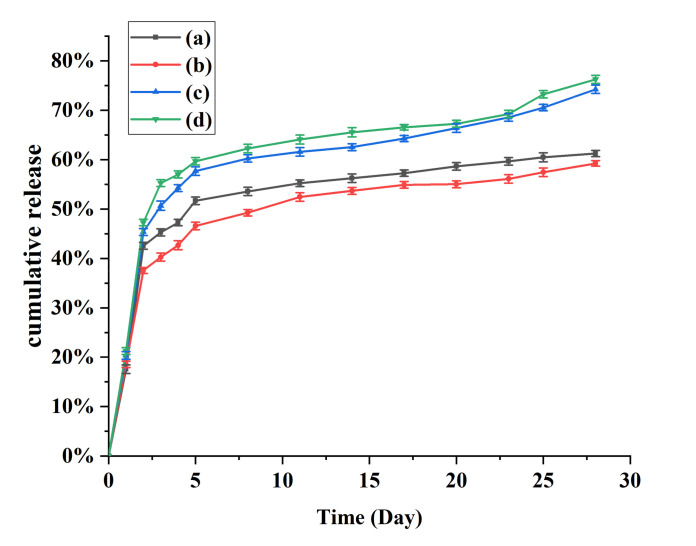
Cumulative release curves for microcapsules prepared under different conditions. (**a**) Internal phase/oil phase = 1:5; oil phase/external water phase = 1:5; (**b**) Internal phase/oil phase = 1:5; oil phase/external water phase = 1:7; (**c**) Internal phase/oil phase = 1:7; oil phase/external water phase = 1:5; (**d**) Internal phase/oil phase = 1:7; oil phase/external water phase = 1:7.

The sustained release properties are an important indicator for evaluating the performance of drug carrier systems. It can be seen from the results in Figure 6 that the cumulative release curves of the microspheres could be divided into three stages: initial burst release, slow release, and long-term sustained release. Within two days of the initial release, the four microcapsule types all exhibited a clear burst effect. The ratio of the internal water phase to the oil phase was found to give the greatest impact on the burst effect. The burst releases of microcapsules prepared with the internal water phase/oil phase ratio of 1:7 were 45.36% and 47.26%, which were higher than the releases exhibited by capsules prepared with internal water phase/oil phase ratio of 1:5, which were 42.56% and 37.56%. This may be owing to the high encapsulation efficiency of microcapsules prepared with the internal water phase/oil phase ratio of 1:7, and because the large difference in drug concentration between the microcapsules and the external environment promoted the release of a large amount of protein from the microcapsules. In terms of the release after the burst effect ends, the cumulative release rate from microcapsules prepared with an internal water phase/oil phase ratio of 1:5 was approximately 58% at 28 days, which is considerably lower than the 77% observed for microcapsules with an internal water phase/oil phase ratio of 1:7. This indicates that microencapsulated ovalbumin formed with a low oil phase ratio was released more slowly and the release lasted longer.

### 3.8. Preparation of Porous PLGA Microcapsules

**Figure 7:**
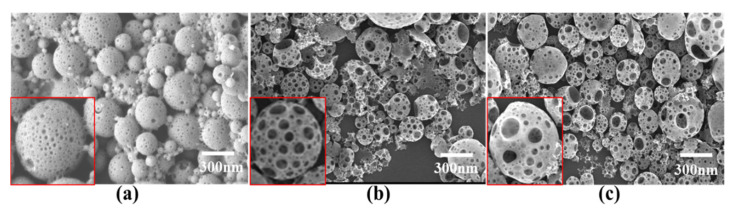
Scanning electron micrographs of porous PLGA microcapsules. (**a**) 10% BSA solution; (**b**) 15% BSA solution; (**c**) 20% BSA solution.

Generally, the capsule material of microcapsules is relatively strong and dense, which leads to unsatisfactory release of the core material. Porous microcapsules have a larger specific surface area than solid microcapsules, and the release rate can be controlled by adjusting the number of pores. Therefore, they have potential applications in drug delivery, agriculture, catalysis and food technology [28]. The addition of porogen BSA to the inner water phase was favorable for forming porous microcapsules. Under the osmotic pressure, water molecules in the external water phase are inclined to transfer into the inner water phase and form a water zone in the oil phase. As the organic solvents evaporate and oil droplets solidify, the retained water zone would form pores in the microcapsules after drying [29]. We investigated the best preparation conditions for PLGA microcapsules and obtained microcapsules with a high encapsulation efficiency, a small particle size, and uniformity. Under these optimal conditions, we prepared porous PLGA microcapsules by adding an appropriate amount of bovine serum albumin to the internal water phase. As shown in Figure 7, as the proportion of BSA solution in the internal water phase increased, the diameter of the pores on the microcapsule surface increased, the particle size of the porous microcapsules did not increase, but the uniformity was affected. More pores on the microcapsule’s surface would accelerate the penetration and release of the loaded drugs due to a larger surface area. As water molecules move into the inner, this also results in the formation of a hollow cavity that is bigger than the microcapsules [30].

### 3.9. Effect of BSA Volume on the Encapsulation Efficiency of PLGA Microcapsules

The volume of BSA solution not only affected the surface pore size and quantity of porous microcapsules, but also their encapsulation efficiency. The encapsulation efficiency of microcapsules measured using the BCA method is shown in Table 7. The addition of BSA solution reduced the encapsulation efficiency of the microcapsules and, as the added volume increased, the decrease became more pronounced. This may be due to the formation of pores allowing protein to diffuse into the external aqueous phase, but the microcapsules still maintained an encapsulation rate of more than 50%.

### 3.10. Effect of BSA Volume on the Sustained Release of PLGA Microcapsules

**Figure 8:**
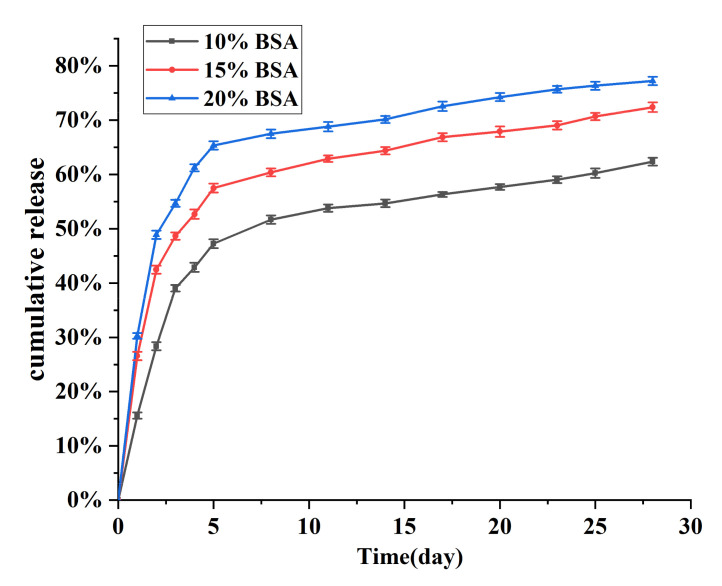
Cumulative release curves for porous microcapsules.

In recent years, to meet various application requirements, different approaches for regulating the release of cargo from microcapsules have been attracting increasing attention. Among the investigated approaches, adjusting the porosity of microcapsules is a simple and effective method [30]. Therefore, we prepared three types of porous microcapsules with different surface porosities by adding different amounts of BSA solution, and evaluated their sustained release. The results are shown in Figure 8. In general, the release curves of the porous microcapsules show the same release behavior as described above for the solid particles. As the amount of BSA increased, the burst release rate of the microcapsules rose. When the amount of BSA added was 10%, the duration of the burst release phase increased from one to two days. In terms of the release stage after the burst effect ended, the three different microcapsules showed a similar release rate from the fifth day onwards, and the cumulative release rates at 28 days were 77.68%, 72.03%, and 62.34% for the 20%, 15%, and 10% BSA solution capsules, respectively. These results indicate that the increase in pore size was the main factor that promoted the release from microcapsules, and the sustained release of the microcapsules could be controlled by adjusting the size of the pores.

## 4. Conclusions

The use of nanoparticles as carriers for drug delivery has been extensively studied. Nanoparticles can prevent the premature degradation of drugs, increase drug uptake and reduce toxic side effects [16]. In addition, the sustained release and targeting properties of nanoparticles can prolong the therapeutic effect and allow drugs to be transported through biological barriers and reach a specific site of action [29]. As a capsule material with good biocompatibility, PLGA has been widely applied in the field of biomedicine; however, the particle size affects the efficiency of drug delivery and absorption, while the thickness of the capsule wall affects their release rate. There are therefore many studies that focus on optimizing the preparation of microcapsules. Various encapsulation techniques have been developed such as double emulsion, in situ polymerization, nanoprecipitation and premix membrane emulsification. It is necessary to obtain microcapsules with a uniform and tailored size for the construction of controlled-release drug carriers [29,30].

In this study, we prepared a variety of PLGA microcapsules by encapsulating ovalbumin using different conditions. The best preparation conditions were selected as follows: concentration of PLGA in the oil phase, 20 mg/mL; concentration of PVA in the external water phase, 5 mg/mL; volume ratio of inner water phase to oil phase, 1:5; volume ratio of oil phase to external water phase, 1:5; and shear rate, 10,000 rpm. The average particle size of the PLGA microcapsules prepared under these conditions was approximately 200 nm, and they showed a uniform size, smooth surfaces without cracks, good dispersion, and no aggregation. The ovalbumin encapsulation rate for these PLGA microcapsules was more than 60%, indicating that they have the potential to be used as protein drug carriers. In addition, to obtain microcapsules with controlled release rates, we prepared PLGA microcapsules with pores on the surface by adding BSA to the internal aqueous phase under the above conditions. The microcapsule pore diameter, and hence the drug release rate, could be precisely controlled by adjusting the concentration of BSA. The porous PLGA microcapsules are therefore expected to be useful carriers for a variety of protein drugs.

This study investigated the optimal conditions for PLGA microcapsules’ preparation and their protein encapsulation as well as their sustained release properties. The experimental results showed that PLGA is an effective protein drug carrier material. In future work, the same method will be used to explore the ability of PLGA to encapsulate and deliver drugs such as DNA vaccines, proteins and antigens, to expand the applications of PLGA carriers. Further research will be essential for discovering the specific mechanisms of this delivery system.

## Figures and Tables

**Table 1:** Effect of PLGA concentration on microcapsule particle size and wall thickness.

PLGA Concentration (mg/mL)	20	40	60
Size(nm)	245 ± 7	631 ± 5	1246 ± 7
Thickness of the microcapsule wall(nm)	25 ± 3	60 ± 6	135 ± 7

**Table 2:** Effect of polyvinyl alcohol concentration on microcapsule particle size.

PVA Concentration (mg/mL)	1	3	5
Size(nm)	648 ± 6	478 ± 4	264 ± 3

**Table 3:** Effect of volume ratio of water phase to oil phase on particle size.

Internal Water Phase/Oil Phase	1:3	1:5	1:7
Size(nm)	458 ± 8	264 ± 6	276 ± 6

**Table 4:** Effect of the volume ratio of the oil phase to the external water phase on microcapsule particle size.

Oil Phase/External Water Phase	1:3	1:5	1:7
Size(nm)	878 ± 9	234 ± 6	203 ± 8

**Table 5:** Effect of shear rate on microcapsule particle size.

Shearing Rate (rpm)	8000	10,000	12,000
Size(nm)	378 ± 4	224 ± 7	257 ± 3

**Table 6:** Effect of each phase volume ratio on the encapsulation efficiency of PLGA microcapsules.

Phase Volume Ratio	Encapsulation Rate
internal water phase/oil phase = 1:5 oil phase/external water phase = 1:5	61.47%
internal water phase/oil phase = 1:5 oil phase/external water phase = 1:7	63.68%
internal water phase/oil phase = 1:7 oil phase/external water phase = 1:5	68.75%
internal water phase/oil phase = 1:7 oil phase/external water phase = 1:7	69.45%

**Table 7:** The effect of different volumes of BSA solution on the encapsulation efficiency of microcapsules.

Proportion of BSA Solution	10%	15%	20%
encapsulation rate	58.23%	55.24%	52.68%

## Data Availability

The data presented in this study are available on reasonable requests from the authors.
